# Development of the Complex General Linear Model in the Fourier Domain: Application to fMRI Multiple Input-Output Evoked Responses for Single Subjects

**DOI:** 10.1155/2013/645043

**Published:** 2013-06-12

**Authors:** Daniel E. Rio, Robert R. Rawlings, Lawrence A. Woltz, Jodi Gilman, Daniel W. Hommer

**Affiliations:** ^1^Section of Brain Electrophysiology and Imaging, LCTS, NIAAA, National Institutes of Health, 10 Center Drive, MSC 1540, Bethesda, MD, USA; ^2^Synergy Research Inc., 12051 Greystone Drive, Monrovia, MD, USA; ^3^Laboratory of Neuroimaging and Genetics, Martinos Center for Biomedical Imaging, Massachusetts General Hospital, Charlestown, MA, USA

## Abstract

A linear time-invariant model based on statistical time series analysis in the Fourier domain for single subjects is further developed and applied to functional MRI (fMRI) blood-oxygen level-dependent (BOLD) multivariate data. This methodology was originally developed to analyze multiple stimulus input evoked response BOLD data. However, to analyze clinical data generated using a repeated measures experimental design, the model has been extended to handle multivariate time series data and demonstrated on control and alcoholic subjects taken from data previously analyzed in the temporal domain. Analysis of BOLD data is typically carried out in the time domain where the data has a high temporal correlation. These analyses generally employ parametric models of the hemodynamic response function (HRF) where prewhitening of the data is attempted using autoregressive (AR) models for the noise. However, this data can be analyzed in the Fourier domain. Here, assumptions made on the noise structure are less restrictive, and hypothesis tests can be constructed based on voxel-specific nonparametric estimates of the hemodynamic transfer function (HRF in the Fourier domain). This is especially important for experimental designs involving multiple states (either stimulus or drug induced) that may alter the form of the response function.

## 1. Introduction

The study of human brain cognitive function has been greatly enhanced by advances made in functional magnetic resonance imaging (fMRI) over the past few decades. The most important technique developed for this purpose utilizes changes in blood oxygen level instigated by stimulus-induced neuronal activation [1]. These changes in blood-oxygen levels produce localized variations in magnetic susceptibility and can be seen in T2*-weighted MRI time series data [2, 3]. These time series data, referred to as blood-oxygen-level-dependent (BOLD) fMRI, typically have a low temporal signal-to-noise-ratio (SNR) [4] as well as high temporal correlation [5] that can make them difficult to analyze. 

Functional MRI data analysis from its initial development has largely been implemented in the time domain [6, 7]. The major temporal focused fMRI analysis software packages are AFNI, SPM, and FSL [7–9] although many other analysis packages are also available and in current use. Generally these temporal domain focused analyses have been extended, to incorporate the statistical methodology of general linear models (GLMs) [8, 10, 11]. While almost exclusively used to analyze group data, GLMs have also been used for individual subject analysis [12]. In either case, the GLM approach requires a number of important assumptions be meant [13], that include foremost that the noise in the time series be independent and identically distributed (i.i.d), that is, ~*N*(0,*σ* **I**). Since BOLD fMRI data has significant autocorrelation, it is necessary to attempt to remove the correlation in the data or incorporate into the GLM analysis a model for the noise that takes this into account. These corrections generally take the form of prewhitening techniques [8, 10], autoregressive (AR) models [14], and restricted maximum likelihood (ReML) methods [15, 16]. However, prewhitening or AR modeling of the fMRI BOLD data has been shown to have limitations [17] and these methods may in many instances only reduce nonwhite residual to about 40% of the total voxels [13, 18]. 

Besides the autocorrelation problem, there are additional major sources of error in modeling BOLD responses that arise in “standard” temporal-based analysis of fMRI time series data. Primarily, *a priori* assumptions as to a general parametric form of the hemodynamic response function (HRF) are often required [19] that could vary over the brain or from experimental conditions [20]. Seldom are these assumptions tested as to their validity for each new experimental design or at every voxel to be analyzed. This incorrect modeling of the HRF can lead to increased variance in the coefficients of temporal-based GLM analyses ultimately affecting the power to detect changes in the BOLD response and in general degrading the validity of the model [21, 22]. Furthermore, it is often the case that additional parametric functions and regression coefficients are typically included in temporal-based GLM models to correct for other perceived confounds. These effects can include those of signal drift, head motion [23, 24], and time shifts errors seen in multislice acquisition of fMRI data [25]. However, it has been stated that even small errors in modeling can result in the loss of statistical power [21], and the inclusion of inappropriate effects can lead to an increase of activated voxels yet reducing the validity of the model [26].

Many of potential sources of error associated with applying the GLM framework in the temporal domain can be eliminated or mediated by implementing the GLM in the Fourier or spectral domain. An important advantage of a Fourier-based methodology [27, 28] is that the statistics at different frequencies are asymptotically independent so that statistical tests in the complex domain, that parallel those for the real domain, can be more easily and directly constructed. In particular, Brillinger [29] developed a spectral domain approach for evoked response experiments that can be adapted to the analysis of single subject BOLD fMRI time series data. In this publication, in response to experimental designs that include repeated measure data, we extend the Fourier-based methodology previously developed to analyze fMRI data for multiple input (stimuli) and single output (one fMRI run). This extension enables us to analyze evoked responses fMRI BOLD data for single subjects that have multiple stimulus inputs and multiple outputs (that is repeated runs fMRI data which we will refer to as “states”). The corresponding mathematical extensions to the theory provide the first full multivariate approach in the Fourier domain of the GLM as applied to evoked response fMRI BOLD data. 

Moreover, as previously mentioned, the use of parametric models for the hemodynamic response function (HRF), somewhat separate from the statistical analysis of fMRI data is another drawback in temporal-based analysis of fMRI data [19, 20] that is naturally addressed in a spectral domain approach. As the Fourier-based GLM incorporates voxelwise nonparametric estimates of the hemodynamic transfer function (HTF) (HRF in the frequency domain) and is focused on hypothesis testing of this estimation. Additional positive consequences of performing hypothesis testing of the HTF in the spectral domain is that signal drift corrections as implemented in the temporal domain are unnecessary as the signal mean differences are not tested; time shift errors are also of no importance since the analysis is carried out in the spectral domain; and finally motion artifacts should be mediated since the BOLD response generally has a spectral power distribution that is different than that for head motion. 

Whereas it should be mentioned that some earlier papers have also used Fourier-domain-based approaches to analyze BOLD fMRI time series data, they have been of limited scope. One of the earliest attempts at a Fourier-based analysis of fMRI was that by Lange and Zeger [30] that focused on the analysis of data obtained from a block experimental design and used a parametric form of the HRF. Another early paper that analyzed fMRI data in the frequency domain was by Marchini and Ripley [31]; however, it was restricted to periodic stimuli. A more recent paper, based on the work by Brillinger [27], is that by Bai et al. [32]. It focused on obtaining unbiased estimates of the HRF using stochastic rather than deterministic input stimuli (the usual design for fMRI experiments). It uses a weighted estimate of the transfer function and appropriate chi-square statistics to analyze sample data from an fMRI experiment with a “simple” design. In contrast, our paper has deterministic inputs or stimuli and an unweighted estimate of the transfer function, an approach that provides estimates with minimum mean square error and focuses on inference testing. Thus, the paper by Bai et al. [32] is attempting to find the best estimate to the transfer function, but not necessarily carrying out multivariate statistical hypotheses testing. Therefore, as previously stated the development in this paper is toward a full “multivariate” approach for hypothesis testing to perform signal detection in the spectral domain using an extension of the general linear model methodology in the complex domain. 

## 2. The General Linear Model in the Fourier Domain for Multivariate Output

### 2.1. Model

Previously, a general linear model in the Fourier, domain to model single or univariate fMRI time series was presented [33–35]. In this model, a simple scalar quantity, **s**(*t*), represented the fMRI time series. In order to model a repeated measures experimental design with multiple fMRI time series for a single subject, the model is extended to incorporate multivariate output as follows. Let

(1) $$\begin{array}{c} s\left(t\right)=\mu +r\left(t\right)*a\left(t\right)+\varepsilon \left(t\right), \end{array}$$

where **s**(*t*) now represents a matrix of size 1 × *S* whose elements consist of multiple (*S*) time series or repeated BOLD fMRI runs for a single subject collected at discrete time points *t* (*t* = 1 ⋯ *T*) and spatial coordinate $\underline{x}=(x,y,z)$ (or voxel position, implicit). ***μ*** is a matrix (size 1 × *S*) whose entries consist of constant values (with respect to time) for each time series. The fixed deterministic input stimuli represented by **r**(*t*) have no spatial dependency. Multiple (*R*) input stimuli require that **r**(*t*) be represented by a 1 × *R* size matrix, and correspondingly the response function **a**(*t*) is represented by a matrix of size *R* × *S*. The symbol ∗ represents a convolution of the entries of the matrix product of **r**(*t*) and **a**(*t*). Thus, each stimulus input type and repeated BOLD fMRI run has a single response function represented by a single matrix entry in **a**(*t*) and calculated at every spatial coordinate $\underline{x}$ (implicit). Each entry of the 1 × *R* matrix **r**(*t*) is a time series having the same length as the collected fMRI time series and consists of 0 s or 1 s at each time point *t*, where a value of 1 represents a stimulus presentation at that time. The error in the data is represented by **ε**(*t*) a matrix of size 1 × *S* and assumes that the noise is stationary with zero mean for each corresponding fMRI BOLD time series collected.

Transforming this model (via the complex Fourier transform) to the frequency domain, we have

(2) $$\begin{array}{c} \tilde{s}\left(\lambda_{k}\right)=\tilde{r}\left(\lambda_{k}\right)\tilde{a}\left(\lambda_{k}\right)+\tilde{\varepsilon }\left(\lambda_{k}\right), \end{array}$$

where *λ*_*k*_ = 2*πk*/*T* and *k* represents the wave or frequency number. $\tilde{a}(\lambda_{k})$, the HRFs representation in the Fourier domain is henceforth referred to as the hemodynamic transfer function or HTF. Periodograms [36] are constructed from the spectral forms of the stimulus input $\tilde{r}(\lambda_{k})$ and BOLD outputs $\tilde{s}(\lambda_{k})$ matrices as follows:

(3) $$\begin{array}{c} I_{\alpha \beta }\left(\lambda_{k}\right)={\left(2\pi T\right)}^{-1}\tilde{\alpha }{\left(\lambda_{k}\right)}^{H}\tilde{\beta }\left(\lambda_{k}\right), \end{array}$$

where *α*, *β* = {**r**, **s**}, *λ*_*k*_ = 2*πk*/*T* and the superscript *H* refers to the Hermitian transpose. Estimates of the cross-spectral functions are then constructed [27] as follows:

(4) $$\begin{array}{c} {\hat{f}}_{\alpha \beta }\left(\lambda \right)={\left(2m+1\right)}^{-1}\sum_{k=-m}^{m}I_{\alpha \beta }\left(\lambda_{k}\right), \end{array}$$

where *λ* denotes the center frequency of a band of frequencies 2*m* + 1 in width and provides stable estimates of the cross-spectral functions. The cross-spectral functions take a slightly different form [27] for the band centered at zero frequency. However, in applications to fMRI time series data, this band is discarded because it includes artifacts (e.g., low-frequency motion drift) and is not used in this paper. The band size chosen is based on statistical power considerations and the spectrum of the input power [34]. 

An estimate of the hemodynamic transfer function (HTF) [27, 34, 37] is given by

(5) $$\begin{array}{c} \hat{A}\left(\lambda \right)={\left[{\hat{f}}_{rr}\left(\lambda \right)\right]}^{-1}{\hat{f}}_{rs}\left(\lambda \right)\;R\times S, \end{array}$$

where the matrix size is included for clarity. Note that a matrix entry ${\langle \hat{A}(\lambda )\rangle }_{ij}$ in (5) contains the HTF associated with the *i*th stimulus input and the *j*th repeated run.

### 2.2. Hypothesis Testing of the Hemodynamic Transfer Function

 Consider the hypothesis

(6) $$\begin{array}{c} H_{0}:Ba\left(\lambda \right)C^{T}=0, \end{array}$$

where **B** is the matrix that allows us to construct hypothesis test for multiple input stimuli and has size *b* × *R*, where *b* can range from 1 to *R*. For example, setting the **B** matrix to the identity matrix **I** (size *R* × *R*) would test whether any input stimuli would evoke a response in the BOLD signal. **C** is a matrix of size *c* × *S* where *c* ranges from 1 to *S*. This matrix allows us to construct hypothesis tests associated with the *S* repeated runs for each subject. For example, taking **B** = [1  0] and **C** = [0   1   0] would test the HTF associated with the BOLD response to the first stimulus type and the second repeated run on a subject. Of particular interest is the case for which the **B** and **C** matrices are identity matrices, of sizes *R* and *S*, respectively. We refer to this case as the omnibus case, that is, *F*_2*bc*;2*h*_(*λ*; **B** = **I**, **C** = **I**) which is related to how well the model generally fits the BOLD fMRI data [34]. 

The test of the null hypothesis (6) takes the form of the following *F*-distribution:

(7) $$\begin{array}{c} F_{2bc;2h}\left(\lambda \right)=\frac{h}{bc}\frac{1-U{\left(\lambda \right)}^{1/d}}{U{\left(\lambda \right)}^{1/d}}=\frac{h}{bc}\left[U{\left(\lambda \right)}^{-1/d}-1\right], \end{array}$$

where

(8) $$\begin{array}{c} d=\{\begin{array}{cc} \sqrt{\frac{b^{2}c^{2}-4}{b^{2}+c^{2}-5}} & b^{2}+c^{2}\neq 5\\ 1 & b^{2}+c^{2}=5, \end{array}\\ h=\left[2\;m+1-R-\left(\frac{c-b+1}{2}\right)\right]d-\frac{bc}{2}+1 \end{array}$$

at each spatial position (implicit) and band (represented by its center frequency *λ*). This *F*-distribution simplifies to a more easily recognizable form [34] in the univariate or single fMRI run case, that is the ratio of the explained (by the HTF) to unexplained variance. 

The construct for the current *F*-distribution is based on Rao's approximation to the *U*-statistics [38]. The form of the complex *U*-statistics for the model presented is based on an extension of the multivariate general linear model [39] from the real to complex domain. It has the following form:

(9) $$\begin{array}{c} U_{2b;2c;2(2\;m+1+b-c-R)}\left(\lambda \right)=\frac{\det G_{c}\left(\lambda \right)}{\det\left[G_{c}\left(\lambda \right)+H\left(\lambda \right)\right]}, \end{array}$$

(10) $$\begin{array}{c} G_{c}\left(\lambda \right)=CG\left(\lambda \right)C^{T},\;c\times c, \end{array}$$

where

(11) G(λ)=(2 m+1)×{f^ss(λ)−f^sr(λ)[f^rr(λ)]−1f^rs(λ)}, S×S,

(12) $$\begin{array}{c} H\left(\lambda \right)=\left(2\;m+1\right)E{\left(\lambda \right)}^{H}{\left[V\left(\lambda \right)\right]}^{-1}E\left(\lambda \right),\;c\times c, \end{array}$$

where

(13) $$\begin{array}{c} E\left(\lambda \right)=B\hat{A}\left(\lambda \right)C^{T},\;b\times c,\\ V\left(\lambda \right)=B{\left[{\hat{f}}_{rr}\left(\lambda \right)\right]}^{-1}B^{T},\;b\times b, \end{array}$$

Factors of 2, associated with the degrees of freedom in (7) and (9), are required to account for the cross-spectral estimations having both real and imaginary parts. Note that all terms in (12) except that for ${\hat{f}}_{rr}(\lambda )$ and correspondingly **V**(*λ*) are implicit functions of the spatial coordinates $\underline{x}=(x,y,z)$ or voxel position.

## 3. Methods

### 3.1. Experimental Design and Data Acquisition

The experiment consisted of the following paradigm. An alcohol-dependent and control subject taken from a larger study using event-related fMRI [40] was investigated. The experiment consisted of obtaining three separate BOLD fMRI images per subject. During each acquisition, which we will henceforth refer to as a state, the exact same visual input stimulus sequence was presented to the subject. These visual images were chosen from the International Affective Picture System [41]. Each image was presented for two seconds with a random interstimulus interval from 0 to 8 sec. Each subject was then asked to evaluate the visual stimuli, having either positive (pos) or negative (neg) valence using one of two buttons available for them to press using three separate criteria (counterbalanced order). 

Specifically, subjects were asked to:

- evaluate the environment of the presented image (whether it is indoor or outdoor) for which the collected BOLD time series is referred to as the cognitive state;
- evaluate the emotional valence of the presented image, if you liked it or did not like it, for which the collected BOLD time series is referred to as the emotion state;
- do not evaluate the image and simply press a button when presented with an image for which the collected BOLD time series is referred to as the passive state.

Henceforth, we will refer to the three BOLD fMRI time series collected for each subject as the cognitive (cog), emotion (emo), or passive (pas) state. 

### 3.2. Experimental Scanning Parameters

Images were collected on a 3T GE MRI scanner (General Electric, Milwaukee, WI, USA), using a standard quadrature head coil. The fMRI scans consisted of 156 temporal volumes (64 × 64 × 16) consisting of 5 mm thick slices with in-plane sampling of 3.75 × 3.75 mm using a T2*-weighted echo-planar sequence with TR = 2 s, TE = 40 ms, and flip angle 30°. Structural scans were acquired using a T1-weighted MP-RAGE sequence with TR = 100 ms, TE = 7 ms, and flip angle 90°.

### 3.3. Image Preprocessing

Preprocessing of the fMRI single subject images consisted of the following steps.

1. Spatial registration of all functional temporal volumes to the tenth-time volume collected in the passive run using the AFNI [7] program 3dvolreg. The AFNI program 3dAutomask was also used to construct a binary mask (inside brain versus outside brain) for the functional images.
2. Within-slice (2-dimensional) spatial smoothing using a Gaussian filter 8 mm full width half-maximum (FWHM) was applied to the coregistered images produced from step 1.
3. Structural-to-functional MRI registration and restricted-to-affine transformations within subject using the AFNI program 3dAllineate [42] were performed. That is, the tenth-time volume or BOLD image from the passive run (see step 1) was registered to the subject's own structural MRI volume image.

Notably no other preprocessing of the data was made (and none was required [34]) in contrast to standard preprocessing of fMRI data in the time domain [23–25, 43].

### 3.4. Analysis of Multivariate Data in the Fourier Domain

All statistical tests were performed on an Apple Mac Pro Dual-Core 2.66 GHz computer using the SRView program (unpublished) developed for general fMRI data analysis. SRView is programmed in C++, with a X11-based GUI with embedded functional calls or a batch mode that uses tcl/tk as a scripting language. C shell scripts can also be used to invoke multiple runs of SRView. Typical calculations that include all hypothesis tests usually take twenty minutes or less.

Statistical tests of the null hypothesis (6) were carried out using the corresponding *F*-statistics (7) with appropriate **B** and **C** matrices chosen for a specific test. A band size of 13 frequencies (*m* = 6) was chosen based on the observed spectral power distributions and from previously analyzed data results [34, 35, 37]. Once a uniform band size was chosen the partitioning of the Fourier frequencies into bands and associated center frequencies was set. The band centered at zero frequency (*λ* = 0) was discarded because it contains a number of low-frequency artifacts. These are most prominently associated with motion and possibly signal drift. Therefore the elimination of this band is equivalent to applying a high-pass frequency filter to the time series data. After discarding this zero-band and limiting the highest frequency band to have an upper bound less than or equal to the Nyquist frequency, we produced five bands of equal size. *F*-statistics (6) were then calculated as a function of the center frequency *λ* of these bands.

Initially, model goodness of fit was explored using the omnibus hypothesis test *H*_0_ : **A**(*λ*) = 0 using the *F*-test statistic (7) with **B** and **C** matrices set as identity matrices. These tests were carried out at every voxel within the scope of the brain masks produced in the preprocessing steps (see Section 3.3). These tests performed at every voxel indicated whether any of the stimuli produced a significant response at any center frequency. The resultant spatial patterns are presented using multiple *P*-level mask (typical for reporting fMRI activity in temporal-based analysis). A color look-up-table (LUT) is used to present these threshold values. The algorithm for production of the multiple *P*-level mask in the Fourier domain is shown in Algorithm 1.

**Figure 1:**
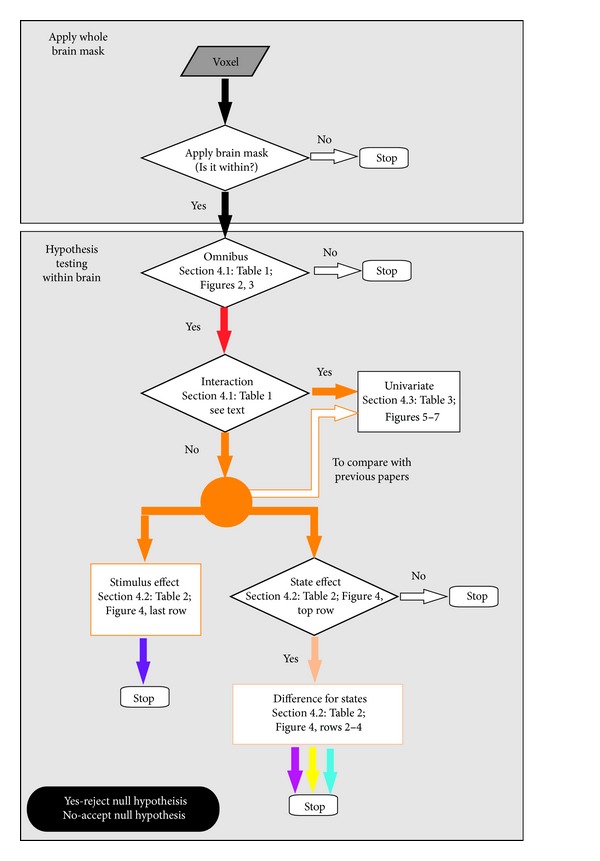
Diagram showing the sequential processing of a voxel in terms of hypotheses test applied. Only voxels for which the null hypothesis is rejected (except in the case of the interaction) proceed to the next test. For a complete description of tests and associated figures, see the indicated sections referred to in the flow chart.

To help control for multiple tests (that is limiting the number of false positives), all voxels originally sampled within the full brain mask were further restricted by statistically based “voxel limiting” spatial masks as follows. The omnibus *F*-test images (related to measure of model fit—see Section 2.2) at each center frequency were strictly threshold at *P* = .001 to produce binary masks for each band. These masks were then combined using a Boolean OR operation to produce one spatial binary mask, henceforth referred to as the omnibus mask. This mask enabled us to limit the number of voxels looked at with the specific inference tests for interaction, main and simple effects for the ANOVA design presented. This yielded approximately 7% of the brain mask voxels for the alcoholic subject. Next, a mask based on the interaction hypothesis test was produced. The test for interaction between stimuli and states produced a mask used to exclude those voxels in which an interaction was seen. Additional multivariate tests for state effects were also used to further spatially restrict subsequent hypothesis tests. The algorithm for application of these hierarchical embedded masks is presented in Figure 1, where we present the flow chart associated with a voxel as it is either included or excluded in a mask, whose construct is based on the specific criteria being tested. Note that less stringent criteria were applied to univariate-based hypothesis tests where only the simple omnibus test based mask was applied so as to more easily compare the results to the previously published analysis of this data [40].

Finally, in this analysis no attempt was made to investigate the frequency structure of the response since the temporal sampling rate or TR was relatively long and only a few bands were available for testing. For a more detailed look at frequency-specific hypothesis testing, see Rio et al.'s work [34] where the TR was 400 ms and the acquired fMRI series were 1400 time points long.

## 4. Results and Discussion

### 4.1. Omnibus Hypothesis Test and Test for Interaction

**Figure 2:**
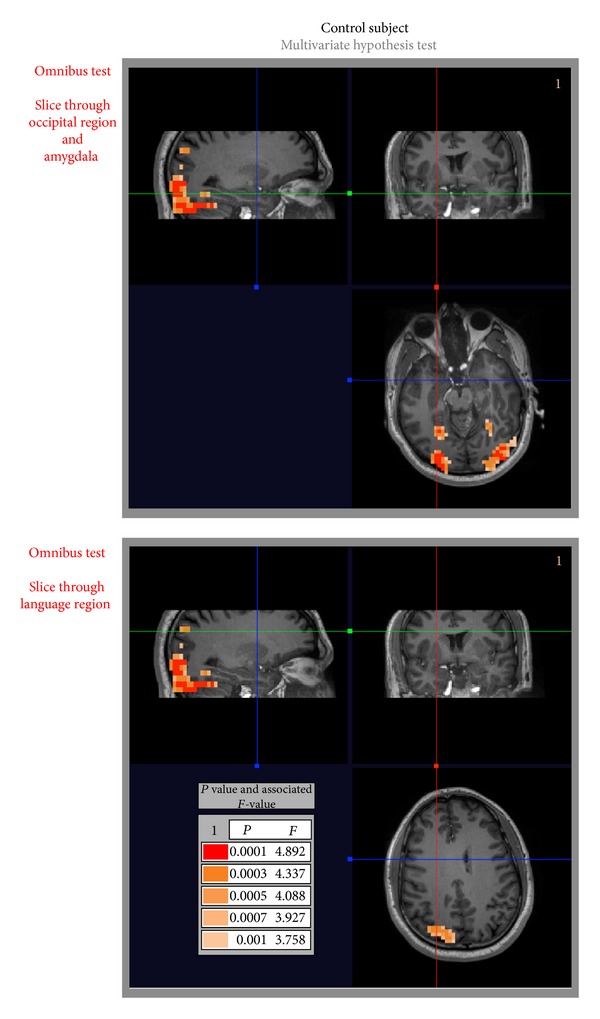
Control subject: inference results for omnibus hypothesis test shows BOLD response in the occipital region of the brain, visual stream. Note that the red blue and green lines indicate the correspondence between the orthogonal slices presented. There is no activation in the language region for the slice presented or any other slice through the language regions (not presented).

**Figure 3:**
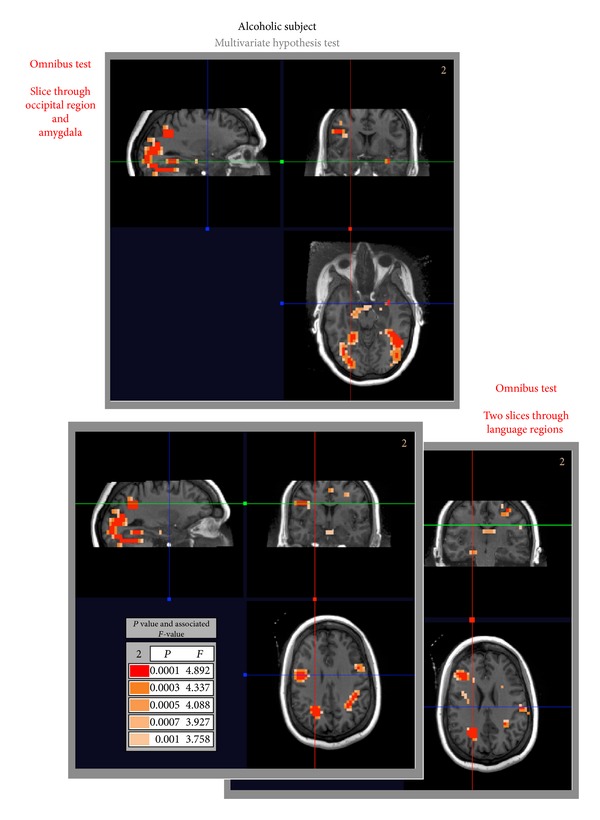
Alcoholic subject: inference results for omnibus hypothesis test shows BOLD response in the occipital region of the brain, visual stream. Note that the red blue and green lines indicate the correspondence between the orthogonal slices presented. Also interestingly, the amygdala and language areas for the two slices presented show a BOLD response not seen in the control subject (Figure 2).

The construction of the omnibus hypothesis test for the control and alcoholic subjects is performed first. This consisted in applying the *F*-test (6) with full-rank matrices **B** and **C** set to **I** to test the hypothesis of whether any input stimuli or output run produced a significant response.  Table 1 lists the test, and selected image slices (showing relevant results) are presented in Figures 2 and 3. In both figures, we see significant activation in the occipital regions of the brain that can generally be attributed to the visual stimuli being processed. Additionally, for the alcoholic subject, we see strong activation in the languages regions (both Broca's and Wernicke's areas—bottom of Figure 3) as well as some more muted activation (associated with a larger *P* value) in the amygdala (top of Figure 3). No such activation occurs in the control subject in the language area or in the amygdala (Figure 2).

A test for interaction was also performed (see Table 1). This test for parallel profiles based on the differential response of positive to negative stimuli for the output vector of cognitive, emotional, and passive states. This produced only a few activated voxels (not shown in any figure) in the control and alcoholic subjects. Most notably in the alcoholic subject a small loosely connected set of three voxels in the left insula region that was not seen in the control subject. It is interesting to note that this is an important region of the brain linked to emotion and cognitive functioning. 

### 4.2. Multivariate Repeated Measures Hypothesis Testing

**Figure 4:**
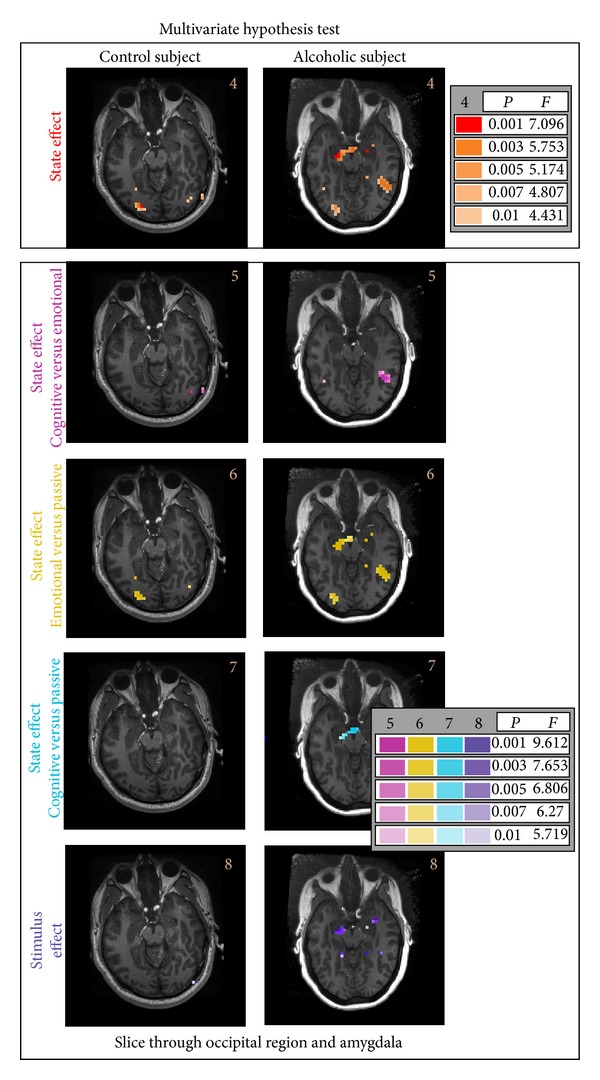
Multivariate repeated measures hypothesis tests for the control and alcoholic subjects are presented for a slice representative of the amygdala (media anterior) in the brain. The hypothesis tests show significant BOLD activation in the alcoholic subject in the amygdala for both a state effect and stimulus effect. Differences between the cognitive, emotional and passive states are also seen. Finally, while no direct hypothesis test between subjects can be made, we see in the control subject generally a more subdued BOLD response in all cases.

The main effects were investigated using multivariate repeated measures hypothesis tests (see Table 2). The images presented in Figure 4 are those for a control and alcoholic subjects for transversal slices that cut through the occipital region of the brain posteriorly and through the amygdala in the medial anterior. The first hypothesis tested is that for the state effect, that is, whether the stimulus inputs, positive and negative, showed a differential response in the state vector consisting of the cognitive, emotional, or passive functional MRI runs. The degrees of freedom for the *F*-distribution used in this test are (4, 20) (with *b* = 1, *c* = 2; see Table 2, Test no. 4 and (7)). Next are presented the results for the following hypotheses: the effect between the cognitive and emotional states; the effect between the emotional, and passive states and the effect between the cognitive and emotional states. Finally, the hypothesis for stimulus effect is presented, that is, whether a differential response was seen between positive and negative input stimuli across the state vector, that is, the cognitive, emotional, or passive functional MRI runs. The degrees of freedom for the *F*-distribution used in these test are (2, 22) (with *b* = 1, *c* = 1; see Table 2, Tests nos. 5–8 and (7)). Generally, it is seen in Figure 4 that the alcoholic subject shows a pattern of BOLD response not seen in the control subject for any of the hypotheses tested, especially in the amygdala (medial anterior brain structure). However, no direct between-subject inference test is available for the single subject analysis presented. 

### 4.3. Univariate Simple Effects Hypothesis Testing

**Figure 5:**
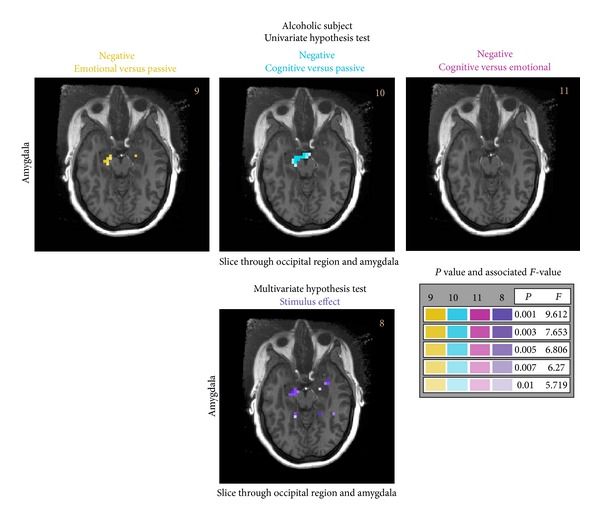
Univariate simple effects hypothesis test results are presented for the alcoholic subject for a slice representative of the amygdala (media anterior) in the brain. Focusing on the negative stimulus input and comparisons between the three output states, that is, emotional versus passive states, cognitive versus passive states, and cognitive versus emotional states. For context, an additional hypothesis test result (also see Figure 4) is presented showing the stimulus effect.

The remaining hypothesis tests to be presented are simple effects tests (see Table 3). In Figure 5, we focus generally on comparisons between the output states in the amygdala, that is, the differential responses between the emotional, passive, and cognitive states for the negative stimuli input in the alcoholic subject. The largest (that is spatially extended region) differences occur between the emotional and passive states or the cognitive and passive states in this alcoholic subject. The stimulus effect hypothesis (bottom row of Figure 5 and also presented in Figure 4) also shows a differential response between the positive and negative stimuli in this same region. 

**Figure 6:**
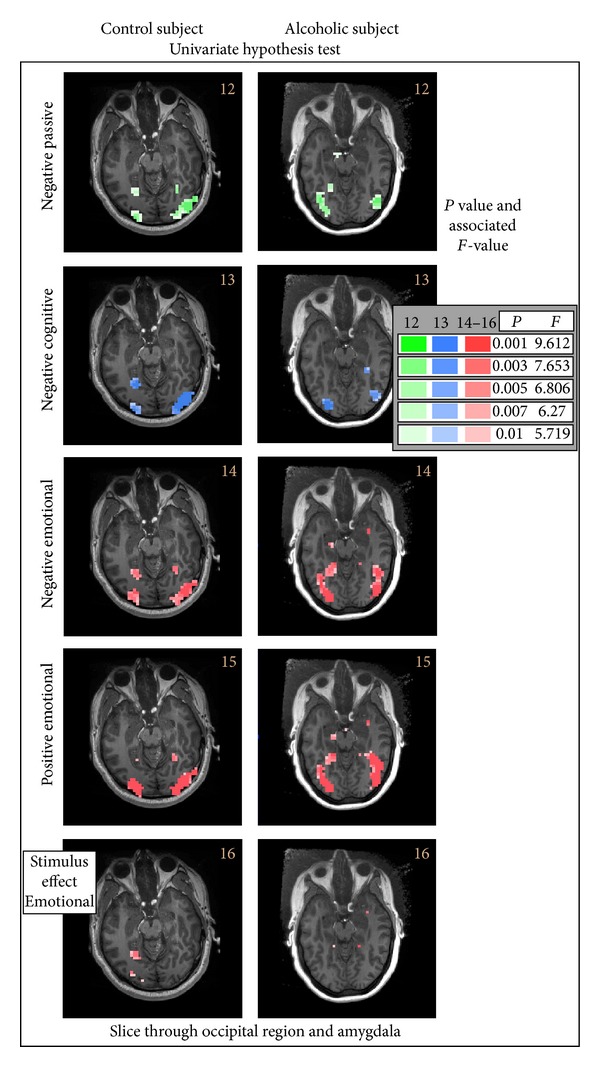
A sampling of univariate simple effects hypothesis test results at a slice level that includes the amygdala for the control and alcoholic subjects. Focusing on the negative stimulus input for the three states (repeated BOLD fMRI image runs), passive, that is, cognitive, and emotional in the first three rows. BOLD response is most extensive in the emotional state. The fourth row shows the positive stimulus, emotional state BOLD response. Finally, in the last row is presented the stimulus response for the emotional state. See Table 3 for a description of all the matrices used to test these hypotheses.

We next present some simple effects of hypothesis test results for both the control and alcoholic subjects in Figure 6 that again present results on a transversal slice that includes the amygdala. These hypothesis tests, testing simple effects associated with one or the other stimulus input and one of the output states, passive, cognitive, or emotional, show minimal activation except possibly for the emotional state. In Figure 6 (last row) is presented the activation mask associated with the stimulus effect, that is, the differential response of the input stimuli in the emotional run, which shows some differential response to the stimuli in the amygdala.

**Figure 7:**
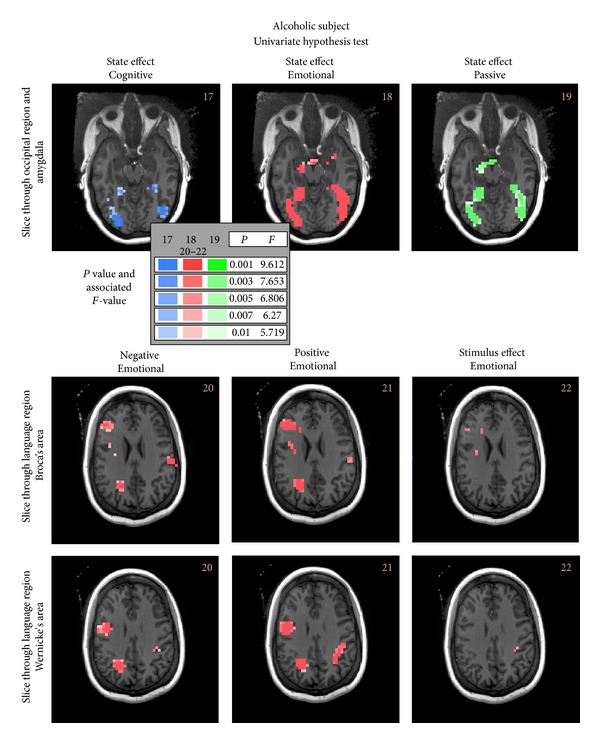
Univariate simple effects hypothesis tests results for the alcoholic subject at three slice levels that include the amygdala and the language areas of the brain are presented. Particularly note the increased activation shown in the emotional state in the amygdala. On the bottom two rows are presented: the hypothesis tests for the emotional state for each individual input stimulus as well as the stimulus effect through slices that include both Broca's and Wernicke's language areas of the brain. The resultants indicate that the emotional state was the major contributor to the omnibus hypothesis test results shown in Figure 3 in the language area.

Finally, in Figure 7 are presented the results for simple effects univariate hypothesis tests. They include hypothesis tests for the negative, positive, or stimulus effect (differential response between the inputs, positive and negative stimulus inputs) for the cognitive state, emotional state and passive state for the alcoholic subject in both the amygdala and language areas of the brain. Here, we see increased activation in the emotional and passive runs as compared to the cognitive run in the amygdala and the occipital region. Particularly in the language regions, both Broca and Wernicke's (bottom two rows of images in Figure 7), we see substantial activation for the positive or negative stimuli and a somewhat smaller activated region for the stimulus effect (that is differential test between input stimuli) for the emotional run. The cognitive and passive runs were either not as active or generally inactive for these simple effects hypotheses tests and are not presented.

### 4.4. Hypothesis Testing in the Temporal Domain: A Sample Result from a Previous Study

**Figure 8:**
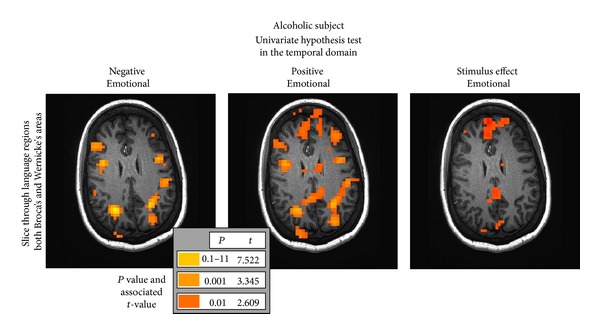
Temporal *t*-based statistical hypothesis test results for the emotional run in the same alcoholic subject analyzed in the Fourier domain (Figure 7, last two rows—Tests nos. 20–22). This exact single subject analysis was incorporated into the group of alcoholics used to produce a major result in the original paper by Gilman et al. [40]. Activation in both Broca's and Wernicke's language areas of the brain is shown in this single slice as well as a number of other regions of less interest.

A previous temporal-based analysis result for a slice that includes both Broca's and Wernicke's language areas of the brain for the alcoholic subject used in our Fourier-based fMRI analysis is presented. The control subject's result is not presented since our omnibus hypothesis test results (Figure 1) showed no activation in this region. The result presented here is the exact single subject analysis incorporated into the group analysis by Gilman et al. [40] employed to produce a major result of that paper (see Section 5). Comparison to the full group results is beyond the scope of this paper; however, a qualitative comparison can be made to the single subject alcoholic subject used in both papers. The single slice (Figure 8) covers the same anatomical regions as the slices presented in the two bottom rows of Figure 7. Activation is seen in the language area of the brain, similar to that produced in the group results [40] as well as in other regions of lesser interest in this particular subject. While no straightforward comparison of the *F*-statistic result (Figure 7, bottom two rows) and the *t*-statistics result (Figure 8) is possible, multiple *P* value spatial results are shown in both figures. Note that a region presented with a value of .001 means that this includes all voxels in which the statistic had a threshold in which *P* < .001. Using this as a guide, it is possible to observe similar language regions seen in both analyses; however, the temporal-based analysis seems to be more generous in its assignments of activated region based on *P* values tested, especially in the hypothesis test results for the positive stimuli. This also gives rise to activated regions for the stimulus effect (last image on the right) not seen in the Fourier-based analysis method. The additional regions of activation are typical in temporal-based analysis that use less general forms for the noise error [13] and are not an indication of increased sensitivity with these tests. In fact, the group analysis using this subject corrects this problem where many of these activated regions are no longer significant. On the other hand, the language region for the emotional run, as seen in the Fourier method, is one of the major activated regions in the temporal-based group analysis that turns out to be important. 

## 5. Conclusion

Extensions have been developed and presented to the complex general linear model with multiple inputs and outputs that provides a statistically rigorous methodology to analyze fMRI time series data for single subjects based on the theory developed by Brillinger [27, 29]. In doing so, we have incorporated the standard notation of the general linear model in the real domain as presented by Timm [39] for multiple subjects and adapted it to the case of spectral bands. This approach allows the stochastic portion of the data to be modeled by a more general form for the noise and therefore fewer restrictions on the structure of the covariance matrix as compared to current time-based analyses. This is especially important in the analysis of single subject data where the assumptions on the noise structure can be critical to the calculation of the accompanying statistics [13]. 

This methodology inherently incorporates voxel-specific nonparametric estimation of the hemodynamic transfer functions (hemodynamic response function in the time domain) that are central to the inference testing procedure. Thus, this methodology is centered on hypothesis testing of this transfer function for all constructed multivariate or univariate tests and does not require separate and possibly problematic *a priori* assumptions for the form of the hemodynamic response function as often required in time-based fMRI analysis [13, 19, 20]. In particular, the lack of a requirement to make *a priori* assumptions about the hemodynamic response function form makes this method particularly useful in experiments designs where either drugs or the experimental manipulation itself may alter the form of the hemodynamic response function. This can, for instance happen with the introduction of a vasoactive drug, such as alcohol, to a subject during part of an experimental procedure [20]. 

Finally, by limiting the number of preprocessing steps and/or regressors, our Fourier-based GLM approach should mediate or eliminate many potential sources of error for single subject analysis addressed in the paper by Monti [13].

In regard to the comparison of this Fourier domain approach to the usual temporal-domain-based analyses, we can say the following. Hypotheses tested by these two methods are entirely different. In the temporal domain, a parametrically defined HRF family of functions is used to produce regressors associated with the stimulus input. Typically, amplitudes of the associated HRFs for specific stimuli provide the regression coefficients. Other regressors of noninterest are included for motion, and detrending of the time series data is also performed in this methodological approach. Statistics are then constructed to test the null hypothesis, essentially that the coefficient of the stimuli associated HRFs are zero. In contrast, the Fourier-based method for single subject hypothesis tests completely different. We focused on comparing the entire shape of the HTFs rather than simply comparing the amplitude of HRFs with a similar underlying shape as is often done in the temporal domain. This is accomplished by constructing hypothesis tests is directly on the HTF shape as represented by its spectral profile. Here voxel-specific HTFs are estimated as a direct response to the stimuli presented. That is, a spatially varying measure of the response to the stimuli is presented by the hemodynamic system associated with the brain. The specific and general advantages to this approach are mentioned in previous paragraphs.

From an experimental design chosen to demonstrate the use of this methodology, we have presented the analysis of a single control and alcoholic subject. This design incorporated multiple visual stimuli input and acquired multiple-output state fMRI data. While not rigorously comparable, we see that this analysis shows similar regions of BOLD response to those seen in the original temporal-based group analysis of this data. This can best be summed up by quoting from the conclusion of the original paper by Gilman et al. [40], “Alcoholic patients appear to use brain language areas more than non-alcoholics while making judgments about the setting or liking of emotionally arousing visual images. This increased activation may reflect a compensatory recruitment of brain regions to perform simple decision-making tasks.” Of additional importance, the choice of this experimental design for use in our demonstration has also allowed us to present a systematic approach to avoiding bias in multivariate hypothesis testing by incorporating hierarchical embedded restricting masks. This is an important step in controlling the number of false positives in multivariate-based analysis of fMRI imaging data. Planned extensions would also incorporate a method of false discovery proportion across voxels to further enhance results.

In conclusion, the results obtained from this analysis provide additional confirmation that this methodological approach, previously applied to an experimental design with multiple input stimuli and one output with a fast sampling rate (TR = 400 ms) and Poisson's distributed stimuli [34] can be applied to an experimental design with a more typical design matrix and slower sampling rate (TR = 2 s) that also incorporates multiple (or repeated) fMRI runs for each subject. Having now incorporated multiple input and output hypothesis testing into the Fourier-based GLM approach, this paper provides a foundation to extend this development to the analysis of subject groups in the Fourier domain. Finally while group-based extensions to this methodology will be presented in future publications, let us end by quoting a comment made by Savoy [44], “That ironically it may someday turn out that the information from a few brains, thoroughly studied, will reveal more about universal aspects of human brain function and organization than the current torrent of studies from large collections of brains.” 

## Figures and Tables

**Algorithm 1:**
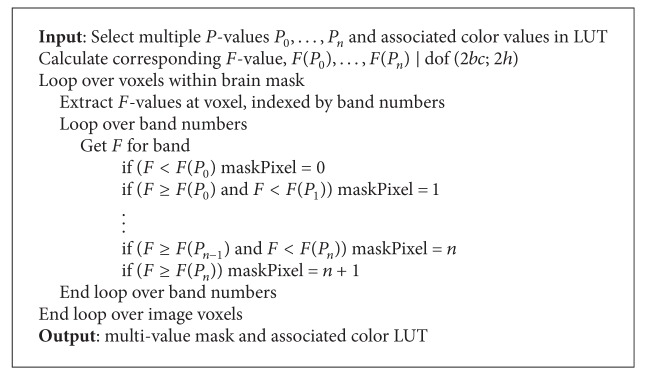


**Table 1:** Compilation of omnibus and interaction hypothesis tests and associated matrices with resultant figures.

Test no.	Subject type slices presented	Hypothesis test	Hypothesis matrices		Figure no.
			**B**	**C**	
1	Subj: control Slices: occipital, language	Omnibus	**I** (identity matrix)	**I**	2
2	Subj: alcoholic Slices: occipital, language	Omnibus	**I**	**I**	3

3	Subj: control, alcoholic Slices: all	Interaction	$\left[\begin{array}{cc} 1 & -1 \end{array}\right]$	$\left[\begin{array}{ccc} 1 & -1 & 0\\ 0 & 1 & -1 \end{array}\right]$	See text

**Table 2:** Compilation of multivariate repeated measures hypothesis tests and associated matrices with resultant figures.

Test no.	Brief description of hypothesis test	Hypothesis matrices		Figure no.
		**B**	**C**	
4	State effect differentiation between states	$\left[\begin{array}{cc} 1 & 1 \end{array}\right]$	$\left[\begin{array}{ccc} 1 & -1 & 0\\ 0 & 1 & -1 \end{array}\right]$	4

5	State effect: cog versus emo	$\left[\begin{array}{cc} 1 & 1 \end{array}\right]$	$\left[\begin{array}{ccc} 1 & -1 & 0 \end{array}\right]$	4
6	State effect: emo versus pas	$\left[\begin{array}{cc} 1 & 1 \end{array}\right]$	$\left[\begin{array}{ccc} 0 & 1 & -1 \end{array}\right]$	
7	State effect: cog versus pas	$\left[\begin{array}{cc} 1 & 1 \end{array}\right]$	$\left[\begin{array}{ccc} 1 & 0 & -1 \end{array}\right]$	

8	Stimulus effect differentiation between stimuli	$\left[\begin{array}{cc} 1 & -1 \end{array}\right]$	$\left[\begin{array}{ccc} 1 & 1 & 1 \end{array}\right]$	4

**Table 3:** Compilation of univariate hypothesis tests and associated matrices with resultant figures.

Test no.	Subject type; slices presented	Hypothesis test	Hypothesis matrices		Figure no.
			**B**	**C**	
9	Alcoholic; occipital	Neg; emo versus pas	$\left[\begin{array}{cc} 0 & 1 \end{array}\right]$	$\left[\begin{array}{ccc} 0 & 1 & -1 \end{array}\right]$	5
10	Alcoholic; occipital	Neg; cog versus pas	$\left[\begin{array}{cc} 0 & 1 \end{array}\right]$	$\left[\begin{array}{ccc} 1 & 0 & -1 \end{array}\right]$	
11	Alcoholic; occipital	Neg; cog versus emo	$\left[\begin{array}{cc} 0 & 1 \end{array}\right]$	$\left[\begin{array}{ccc} 1 & -1 & 0 \end{array}\right]$	

12	Control, alcoholic; occipital	Neg; pas	$\left[\begin{array}{cc} 0 & 1 \end{array}\right]$	$\left[\begin{array}{ccc} 0 & 0 & 1 \end{array}\right]$	6
13	Control, alcoholic; occipital	Neg; cog	$\left[\begin{array}{cc} 0 & 1 \end{array}\right]$	$\left[\begin{array}{ccc} 1 & 0 & 0 \end{array}\right]$	
14	Control, alcoholic; occipital	Neg; emo	$\left[\begin{array}{cc} 0 & 1 \end{array}\right]$	$\left[\begin{array}{ccc} 0 & 1 & 0 \end{array}\right]$	
15	Control, alcoholic; occipital	Pos; emo	$\left[\begin{array}{cc} 1 & 0 \end{array}\right]$	$\left[\begin{array}{ccc} 0 & 1 & 0 \end{array}\right]$	
16	Control, alcoholic; occipital	Stimulus effect; emo	$\left[\begin{array}{cc} 1 & -1 \end{array}\right]$	$\left[\begin{array}{ccc} 0 & 1 & 0 \end{array}\right]$	

17	Alcoholic; occipital	State effect-cog	$\left[\begin{array}{cc} 1 & 1 \end{array}\right]$	$\left[\begin{array}{ccc} 1 & 0 & 0 \end{array}\right]$	7
18	Alcoholic; occipital	State effect-emo	$\left[\begin{array}{cc} 1 & 1 \end{array}\right]$	$\left[\begin{array}{ccc} 0 & 1 & 0 \end{array}\right]$	
19	Alcoholic; occipital	State effect-pas	$\left[\begin{array}{cc} 1 & 1 \end{array}\right]$	$\left[\begin{array}{ccc} 0 & 0 & 1 \end{array}\right]$	
20	Alcoholic; language	Neg; emo	$\left[\begin{array}{cc} 0 & 1 \end{array}\right]$	$\left[\begin{array}{ccc} 0 & 1 & 0 \end{array}\right]$	
21	Alcoholic; language	Pos; emo	$\left[\begin{array}{cc} 1 & 0 \end{array}\right]$	$\left[\begin{array}{ccc} 0 & 1 & 0 \end{array}\right]$	
22	Alcoholic; language	Stimulus effect; emo	$\left[\begin{array}{cc} 1 & -1 \end{array}\right]$	$\left[\begin{array}{ccc} 0 & 1 & 0 \end{array}\right]$	
